# Targeting multiple genes containing long mononucleotide A-T repeats in lung cancer stem cells

**DOI:** 10.1186/s12967-021-02902-6

**Published:** 2021-05-31

**Authors:** Narumol Bhummaphan, Piyapat Pin-on, Preeyaporn Plaimee Phiboonchaiyanan, Jirattha Siriluksana, Chatchawit Aporntewan, Pithi Chanvorachote, Apiwat Mutirangura

**Affiliations:** 1 Center of Excellence in Molecular Genetic of Cancer and Human Disease, Department of Anatomy, Faculty of Medicine, Chulalongkorn University, King Chulalongkorn Memorial Hospital, Bangkok, 10330 Thailand; 2 Cell-Based Drug and Health Product Development Research Unit, Faculty of Pharmaceutical Sciences, Chulalongkorn University, Bangkok, Thailand; 3 Department of Mathematics and Computer Science, Faculty of Science, Chulalongkorn University, Bangkok, 10330 Thailand; 4 Omics Sciences and Bioinformatics Center, Chulalongkorn University, Bangkok, 10330 Thailand; 5 Department of Pharmacology and Physiology, Faculty of Pharmaceutical Sciences, Chulalongkorn University, Patumwan, Bangkok, 10330 Thailand; 6 Faculty of Medicine, King Mongkut’s Institute of Technology Ladkrabang, Bangkok, 10520 Thailand; 7 Center of Excellence in Applied Biosciences, King Mongkut’s Institute of Technology Ladkrabang, Bangkok, 10520 Thailand

**Keywords:** Cancer stem cells, Lung cancer, Mononucleotide A-T repeats, Hallmark of cancer, Universal target

## Abstract

**Background:**

Intratumour heterogeneous gene expression among cancer and cancer stem cells (CSCs) can cause failure of current targeted therapies because each drug aims to target the function of a single gene. Long mononucleotide A-T repeats are cis-regulatory transcriptional elements that control many genes, increasing the expression of numerous genes in various cancers, including lung cancer. Therefore, targeting A-T repeats may dysregulate many genes driving cancer development. Here, we tested a peptide nucleic acid (PNA) oligo containing a long A-repeat sequence [A(15)] to disrupt the transcriptional control of the A-T repeat in lung cancer and CSCs.

**Methods:**

First, we separated CSCs from parental lung cancer cell lines. Then, we evaluated the role of A-T repeat gene regulation by counting the number of repeats in differentially regulated genes between CSCs and the parental cells of the CSCs. After testing the dosage and effect of PNA-A15 on normal and cancer cell toxicity and CSC phenotypes, we analysed genome-wide expression to identify dysregulated genes in CSCs.

**Results:**

The number of A-T repeats in genes differentially regulated between CSCs and parental cells differed. PNA-A15 was toxic to lung cancer cells and CSCs but not to noncancer cells. Finally, PNA-A15 dysregulated a number of genes in lung CSCs.

**Conclusion:**

PNA-A15 is a promising novel targeted therapy agent that targets the transcriptional control activity of multiple genes in lung CSCs.

**Supplementary Information:**

The online version contains supplementary material available at 10.1186/s12967-021-02902-6.

## Background

Cancer stem cells (CSCs) are the major reason for the devastating effects of cancer [1]. CSCs are critical factors that drive cancer aggressiveness, metastasis and drug resistance [1, 2]. Thus, CSC-targeted cancer therapy is crucial. Thus, developing new drugs that can effectively kill the cell population is not only a promising solution but also very challenging.

Intratumour heterogeneous gene expression among cancer and CSCs can cause a failure of current targeted therapy because each drug aims to target the function of a single gene [3]. Currently, CSCs can acquire resistance to EGFR (Epidermal growth factor receptor) inhibitors, which is an effective therapy for lung adenocarcinoma patients with activating mutations, making effective therapy more difficult [4]. Therefore, a novel CSC-targeted therapy is required.

Long mononucleotide A-T repeats are cis-regulatory transcriptional controls of many genes, and the element increases the expression of many genes in various cancers, including lung cancer [5, 6]. A-T repeats are cis-regulatory elements that regulate gene expression by binding Argonaute proteins (Agos) [5]. Therefore, we hypothesized that disruption of A-T repeat interactions with Agos would dysregulate many cancer and CSC genes and that targeting A-T repeats may dysregulate many genes driving cancer development and activate transcription in several ways [6]. Thus, the binding of A and T repeats could be a targeted therapy for cancer treatment. The transcriptional control of the A-T repeat can be disrupted by a peptide nucleic acid (PNA) oligo containing a long A-repeat sequence[A(15)] [5]. Here, we demonstrated that PNA-A15 has promising potential in lung cancer and CSC targeted therapy by dysregulating multiple cancer and CSC genes.

## Materials and methods

### Cell culture and treatment

Human NSCLC-derived cell lines H460, H23, H292, and A549; human kidney cells HK2 and HEK293; and the periodontal ligament (PDL) fibroblast cells were obtained from the American Type Culture Collection (ATCC®, Manassas, VA, USA). H460, H23, and H292 cells were cultivated in Roswell Park Memorial Institute (RPMI) 1640 medium supplemented with 10% fetal bovine serum (FBS), 2 mM L-glutamine, 100 U/mL penicillin and 100 µg/mL streptomycin. A549, PDL fibroblast, HK2, and HEK293 cells were cultured in Dulbecco’s modified Eagle’s medium (DMEM) supplemented with 10% FBS, 2 mM L-glutamine, 100 U/mL penicillin and 100 µg/mL streptomycin. Cells were incubated in a 37 °C humidified incubator with 5% CO_2_ and were routinely subcultured using a 0.25% trypsin solution with 0.53 mM EDTA. RPMI 1640 medium, DMEM medium, FBS, L-glutamine, penicillin/streptomycin, phosphate-buffered saline (PBS), trypsin and EDTA were obtained from GIBCO (Grand Island, NY). In addition, 3-(4,5-Dimethylthiazol-2-yl)-2,5-diphenyltetrazolium bromide (MTT), DMSO, Hoechst 33342, propidium iodide (PI), and bovine serum albumin (BSA) were purchased from Sigma Chemical, Inc. (St. Louis, MO, USA). Antibodies directed against CD44, ABCG2, ALDH1A1 and β-actin and the respective secondary antibodies were purchased from Cell Signaling (Danvers, MA, USA). Antibodies directed against CD133 were purchased from Cell Applications, Inc. (San Diego, CA, USA). PNA-A15 was synthesized from PANAGENE (PANAGENE, Deajeon, Korea. Cells were treated with either PNA-A15 or scramble (control) for 48 h. The culture medium was replaced by medium containing PNA-A15 or scramble every day. The cells were then collected for microarray, anchorage-independent growth, spheroid formation, flow cytometry, and Western blotting analysis.

### Western blotting

Cells were harvested by centrifugation, and resuspended cells were incubated with lysis buffer (20 mM Tris–HCl (pH 7.5), 1% (v/v) Triton X-100, 150 mM sodium chloride, 10% (v/v) glycerol, 1 mM sodium orthovanadate, 50 mM sodium fluoride, protease inhibitor cocktail (Roche Molecular Biochemical) and 100 mM phenylmethylsulfonyl fluoride) for 30 min on ice. The cellular lysates were collected, and their protein contents were determined using a BCA protein assay kit (Pierce Biotechnology, Rockford, IL, USA). Equal amounts of protein from each sample (80 µg for the CSC marker in the total cell population or 30 µg for other proteins and enriched CSC experiments) were separated by SDS-PAGE and transferred to 0.45-μm nitrocellulose membranes (Bio-Rad). The resulting blots were blocked for 1 h with 5% (w/v) nonfat dry milk in TBST (25 mM Tris–HCl (pH 7.5), 125 mM NaCl and 0.1% (v/v) Tween 20) and incubated with the appropriate primary antibodies at 4 °C overnight. After three washes in TBST, the blots were incubated with horseradish peroxidase (HRP)-conjugated secondary antibodies for 2 h at room temperature. Finally, protein bands were detected using an enhanced chemiluminescence substrate (Supersignal West Pico; Pierce, Rockford, IL, USA) and quantified using the analyst densitometry software package (Bio-Rad).

### Spheroid formation assay

The formation of spheroids was performed under serum-free conditions in an ultralow attachment plate as previously reported [7]. NSCLC-derived H292 and A549 cells were pretreated with PNA-A15 and scramble (5 μM) for 48 h. Then, cells were detached using 1 mM EDTA and suspended into single cells. These cells were grown in a 24-well ultralow attachment plate at a density of 2.5 × 10^3^ cells/well in stem cell media (SCM) (i.e., 0.8% (w/v) methylcellulose-based serum-free medium (Stem Cell Technologies, Vancouver, BC, Canada) supplemented with 20 ng/ml epidermal growth factor (BD Biosciences, San Jose, CA, USA), 20 ng/ml basic fibroblast growth factor and 4 mg/ml insulin (Sigma) for 7 days to form primary spheroids. These primary spheroids were harvested, resuspended as single cells using 1 mM EDTA and cultured in SCM for 14 days in a 24-well ultralow attachment plate to form secondary spheroids.

### RNA extraction and microarray analysis

Total RNA was extracted using TRIzol (Invitrogen Life Technologies, Carlsbad, USA); purity and integrity were evaluated using the ND-1000 Spectrophotometer (NanoDrop, Wilmington, USA) and Agilent 2100 Bioanalyzer (Agilent Technologies, Palo Alto, USA). Total RNA was amplified and purified using the TargetAmp-Nano Labelling Kit for Illumina Expression BeadChip (EPICENTRE, Madison, USA) to yield biotinylated cRNA according to the manufacturer’s instructions. After purification, cRNA was quantified using the ND-1000 Spectrophotometer (NanoDrop, Wilmington, USA). Next, 750 ng of labelled cRNA samples were hybridized to each Human HT-12 v4.0 Expression Beadchip for 18 h at 58 °C according to the manufacturer's instructions (Illumina, Inc., San Diego, USA). Detection of the array signal was performed using Amershamfluorolink streptavidin-Cy3 (GE Healthcare Bio-Sciences, Little Chalfont, UK) following the bead array manual. Arrays were scanned with an Illumina bead array Reader confocal scanner according to the manufacturer's instructions. To investigate whether A and T repeats regulate altered gene expression in spheroid formation, two groups of spheroid H292 and A549 cells were compared with H292 and A549 parental cells. Furthermore, whether PNA-A15 alters the expression of genes containing A and T repeats was also assessed. Two groups of spheroids, H292 and A549 cells, were transfected with PNA-A15 and scramble. Each group consisting of 2 samples was studied. Data were uploaded to the Gene Expression Omnibus (GEO: GSE142616).

The down- and upregulated genes in microarray experiments were identified by CU-DREAM (*P*-value threshold = 0.05) [8]. Microarray samples are shown in Table 1. We intersected the down- and upregulated genes between the two experiments. Next, we searched for A-T repeats around the transcription start sites (TSSs) of those genes [5]. The human genome (GRCh38) was downloaded via Entrez Direct (EDirect) and its software package ncbi-entrez-direct. The chromosomal locations of genes were downloaded from ftp://ftp.ncbi.nlm.nih.gov/gene/DATA/gene2refseq.gz. The genome sequence around a TSS was divided into 200 bins, and each bin was 100 bp in length. Bins 1 to 100 covered 10,000 bp upstream of a gene, whereas bins 101 to 200 covered a 10,000bp intragenic region. In each bin, intact A repeats (length 13–27 bp) were identified, and their lengths were summed. For instance, a bin consisted of three A repeats that were 12, 13, and 15 in length. The length sum of this bin was 12 + 13 + 15 = 40. A repeat that overlapped between two bins contributed to each bin proportionately.

**Table 1:** The microarray samples

	Test	Control
1	GSM4232969: Spheroid H292 48hr_rep1	GSM4232971: H292 48hr_rep1
	GSM4232970: Spheroid H292 48hr_rep2	GSM4232972: H292 48hr_rep2
2	GSM4232973: Spheroid A549 48hr_rep1	GSM4232975: A549 48hr_rep1
	GSM4232974: Spheroid A549 48hr_rep2	GSM4232976: A549 48hr_rep2
3	GSM4232961: Spheroid H292 with PNA-A15 48hr_rep1	GSM4232963: Spheroid H292 with scramble 48hr_rep1
	GSM4232962: Spheroid H292 with PNA-A15 48hr_rep2	GSM4232964: Spheroid H292 with scramble 48hr_rep2
4	GSM4232965: Spheroid A549 with PNA-A15 48hr_rep1	GSM4232967: Spheroid A549 with scramble 48hr_rep1
	GSM4232966: Spheroid A549 with PNA-A15 48hr_rep2	GSM4232968: Spheroid A549 with scramble 48hr_rep2

The data were uploaded to the Gene Expression Omnibus (GEO: GSE142616)

Rep1 and rep2 are biological replicate number 1 or 2

### Cytotoxicity assay

For the cytotoxicity assay, H460, H23, H292, A549, HK2, HEK293 and PDL fibroblast cells were seeded onto 96-well plates at a density of 1 × 10^4^ cells/well and allowed to adhere by incubation overnight. Cells were then treated with various concentrations of PNA-A15 or scramble plasmid (0–40 μM) for 48 h and then analysed for cell viability using the 3-(4,5-dimethylthiazol-2-yl)-2,5-diphenyltetrazolium bromide (MTT) assay. Cells were incubated with 500 µg/ml MTT for 4 h at 37 °C, and the intensity of the formazan product after solubilization in 100 µl DMSO was measured at 570 nm using a microplate reader (Anthros, Durham, NC, USA). Relative cell viabilities were calculated by dividing the absorbance of the treated cells by that of the control cells. The half-maximal inhibition concentration (IC_50_) was determined from four independent experiments using GraphPad Prism 5.0 software (La Jolla, CA).

### Anchorage-independent growth assay

After treatment, cells were subjected to an anchorage-independent growth assay based on colony formation in soft agar. The bottom layer was prepared using a 1:1 (v/v) mixture of RPMI-1640 or DMEM medium containing 10% (v/v) FBS and 1% (w/v) agarose. This mixture was allowed to solidify for 30 min, after which the upper cellular layer composed of RPMI-1640 or DMEM medium with 0.3% (w/v) agarose, 10% (v/v) FBS and 1 × 10^3^ cells/ml was prepared and added. Finally, RPMI-1640 or DMEM medium containing 10% (v/v) FBS was added over the upper layer, and the cells were incubated at 37 °C for 14 days. Afterwards, colony formation was observed and imaged using phase-contrast microscopy (Olympus IX51 with DP70). The relative colony numbers and sizes were calculated relative to those for the untreated cells.

### Flow cytometry

H460, H292, H23 and A549 cells were pretreated with PNA-A15 and scramble (5 μM) for 48 h, and then, cells were plated and allowed to form primary and secondary spheroids as detailed above. At day 14 of the secondary spheroid, spheroids were harvested by centrifugation and suspended into single cells using 1 mM EDTA. The resuspended cells were incubated on ice with a rabbit anti-CD133 antibody for 1 h. Next, the primary antibody was removed, and the cells were washed and incubated for 30 min with an Alexa Fluor 488-conjugated goat anti-rabbit IgG (H + L) secondary antibody (Life Technologies, Eugene, OR, USA). After washing, the fluorescence intensity was determined by flow cytometry using a 488-nm excitation beam and a 519-nm bandpass filter (FACSort; Becton Dickinson, Rutherford, NJ, USA). The mean fluorescence intensity was quantified using CellQuest software (Becton Dickinson).

### Spheroid viability assay

Spheroid viability was determined using a water-soluble tetrazolium salt (WST) surrogate viability assay according to the manufacturer’s instructions (Roche Diagnostic GmbH, Mannheim, Germany). Briefly, at day 14 of secondary spheroid formation, as detailed in the “Single 3D spheroid formation assay” section, the cells were incubated with 10% (v/v) WST-1 at 37 °C for 30 min. The intensity of the formazan product was determined using a microplate reader (Anthros, Durham, NC, USA), and the relative cell viability (%) was calculated as the absorbance of PNA-A15 or scramble-treated cells relative to untreated cells.

### Single 3D spheroid formation assay

For CSC-rich population establishment, the spheroid culture assay used in this study was slightly modified from a previously described method [2, 9, 10]. Cells were seeded onto a 24-well ultralow attachment plate with approximately 2.5 × 10^3^ cells/well in 0.8% methylcellulose-based serum-free medium supplemented with 20 ng/ml epidermal growth factor and 4 mg/ml insulin. The primary spheroids were allowed to form for 7 days. At day 7 of primary spheroid culture, primary spheroids were resuspended into single cells using 1 mM EDTA. Again, 2.5 × 10^3^ cells/well were seeded onto a 24-well ultralow attached plate. Secondary spheroids were allowed to form for 14 days. For the single three-dimensional (3D) spheroid-formation assay, cells were allowed to form primary and secondary spheroids as detailed above. At day 14 of secondary spheroid formation, they were dissociated into a single spheroid of the same size, and each spheroid was then treated with 5 µM PNA-A15 or scramble and allowed to grow in the indicated time. Phase-contrast images of the secondary spheroids were obtained at days 0, 3 and 7 after PNA-A15 or scramble treatment under a phase-contrast microscope (Nikon Eclipse Ts2).

### Nuclear staining assay

At day 14 of secondary spheroid formation, these spheroids were dissociated into a single spheroid of the same size, and each spheroid was then treated with 5 µM PNA-A15 or scramble and allowed to grow in the indicated time. Phase-contrast images of the secondary spheroids were obtained at days 0, 3 and 7 after PNA-A15 or scramble treatment under a phase-contrast microscope. On day 7, every individual spheroid was incubated with 10 μg/ml Hoechst 33342 for 30 min followed by 5 μg/ml PI for 5 min. Nuclear condensation and DNA fragmentation of apoptotic cells were visualized using a fluorescence microscope (Olympus IX5; 40 ×) equipped with a DP70 digital camera system (Olympus, Tokyo, Japan).

### Statistical analysis

Data from three or more independent experiments are presented as the mean ± standard deviation (SD). Multiple comparisons for significant differences between multiple groups were performed using analysis of variance (ANOVA) followed by individual comparisons with Scheffe’s post hoc test. Statistical significance was accepted at the *P* < 0.05 level.

## Results

### A-T repeats regulate altered gene expression in CSC-rich spheroids

**Fig. 1:**
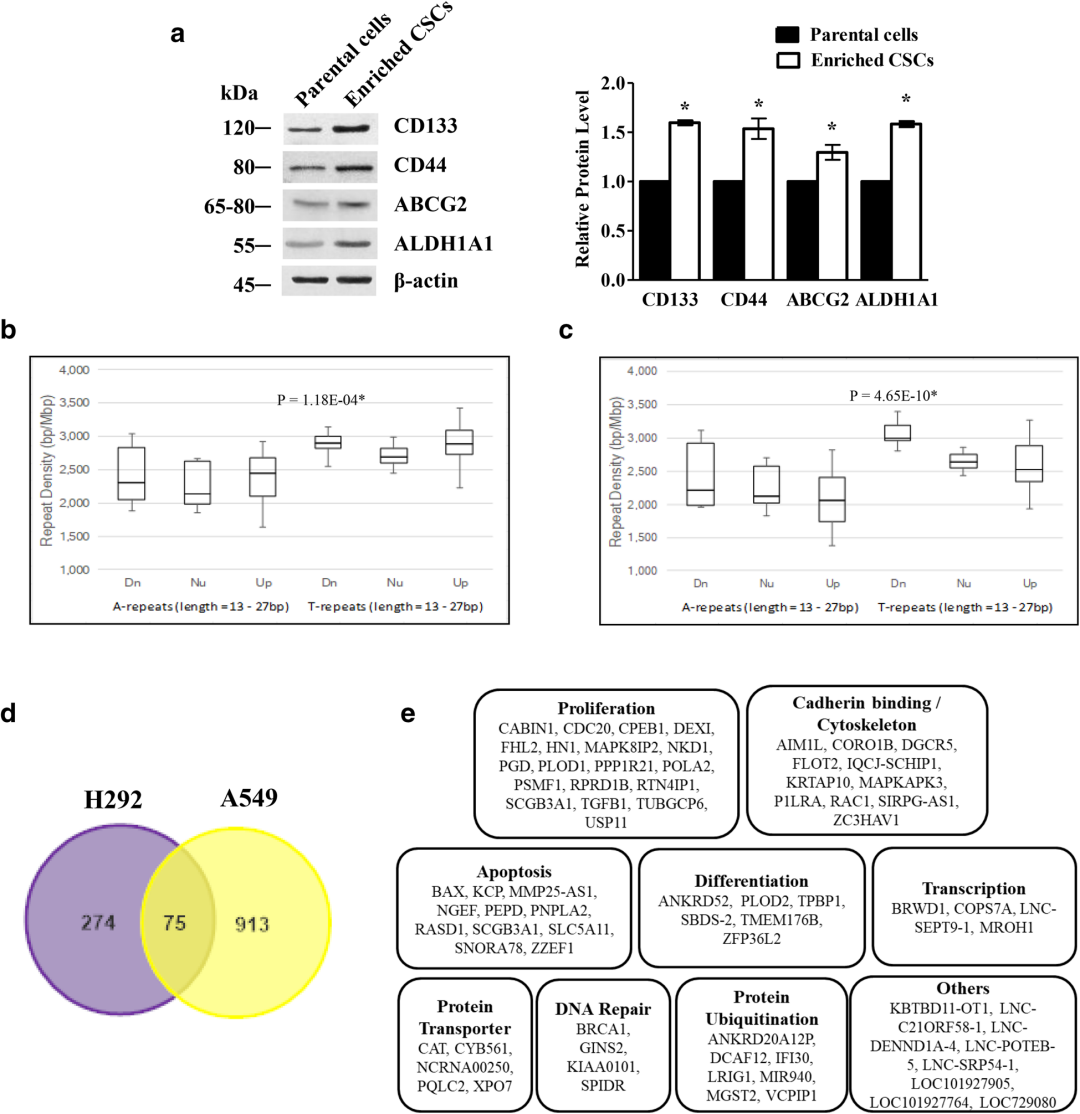
The expression of down- and upregulated genes containing A and T repeats (X-axis) with the repeat density in bp/Mbp (Y-axis). **a** Lysates of parental cells and CSC-enriched cells were prepared and analysed for CD133, CD44, ABCG2 and ALDH1A1 expression by Western blotting. The GSE142616 microarray dataset was downloaded from GEO. Only perfect A and T repeats were classified as downregulated (Dn), neutral (Nu), or upregulated (Up) genes. Experimental and control samples were the spheroid and monolayer of **b** H292 and **c** A549 cell lines, respectively. **d** Venn diagram shows overlap between the intersection of downregulated genes between H292 (the left circle) and A549 (the right circle) cell lines. **e** The list of candidate genes determined by biological process from Gene Ontology. Student's t-test *P*-values, which are denoted by *P*, indicate that the mean difference between Dn/Up and Nu is statistically significant. All plots present the mean ± SD (n = 3). *P < 0.05 vs. untreated cells

CSCs from H292 and parental cell lines were prepared following previous studies [7, 10] and tested for CD133, CD44, ABCG2, and ALDH1A1, which are well-known CSC markers [10, 11]. We found that the expression levels of CSC markers CD133, CD44, ABCG2 and ALDH1A1 were all dramatically increased in the enriched CSC population compared to their parental cells (Fig. 1a).

A previous study demonstrated that genes containing long mononucleotide A-T repeats are overexpressed in various cancers [6]. To determine whether A-T repeats regulate genes differently between lung cancer and CSCs, we compared A-T repeats around TSS to the spheroid formation population of H292 and A549 lung cancer cells. We divided the expression profiling of genes in the spheroid population compared to the total population of their parental cells into 3 groups, up, down, and neutral, depending on the levels of mRNA. The results showed that the T repeat densities of downregulated genes were significantly increased compared with those of neutral genes in the spheroid population of H292 and A549 cell lines (*P*-values = 1.18E-04 and 4.65E-10, respectively). These results suggest that genes containing A-T repeats of cancer cells and CSCs are differentially expressed (Fig. 1b, c).

Then, the candidate genes were selected from overlapping T repeat densities of downregulated genes between H292 and A549 cells and were classified by a biological process based on Gene Ontology analysis as shown in Fig. 1d and Additional file 1: Table S1. Figure 1e shows that the 75 overlapping genes were filtered with a *P*-value ≤ 0.05 in both H292 and A549 cells and were related to genes depending on their functions. We found that dysregulated A and T repeat densities of CSCs in both cell lines were different.

### PNA-A15 is not toxic to normal cells but effectively kills cancer cells

**Fig. 2:**
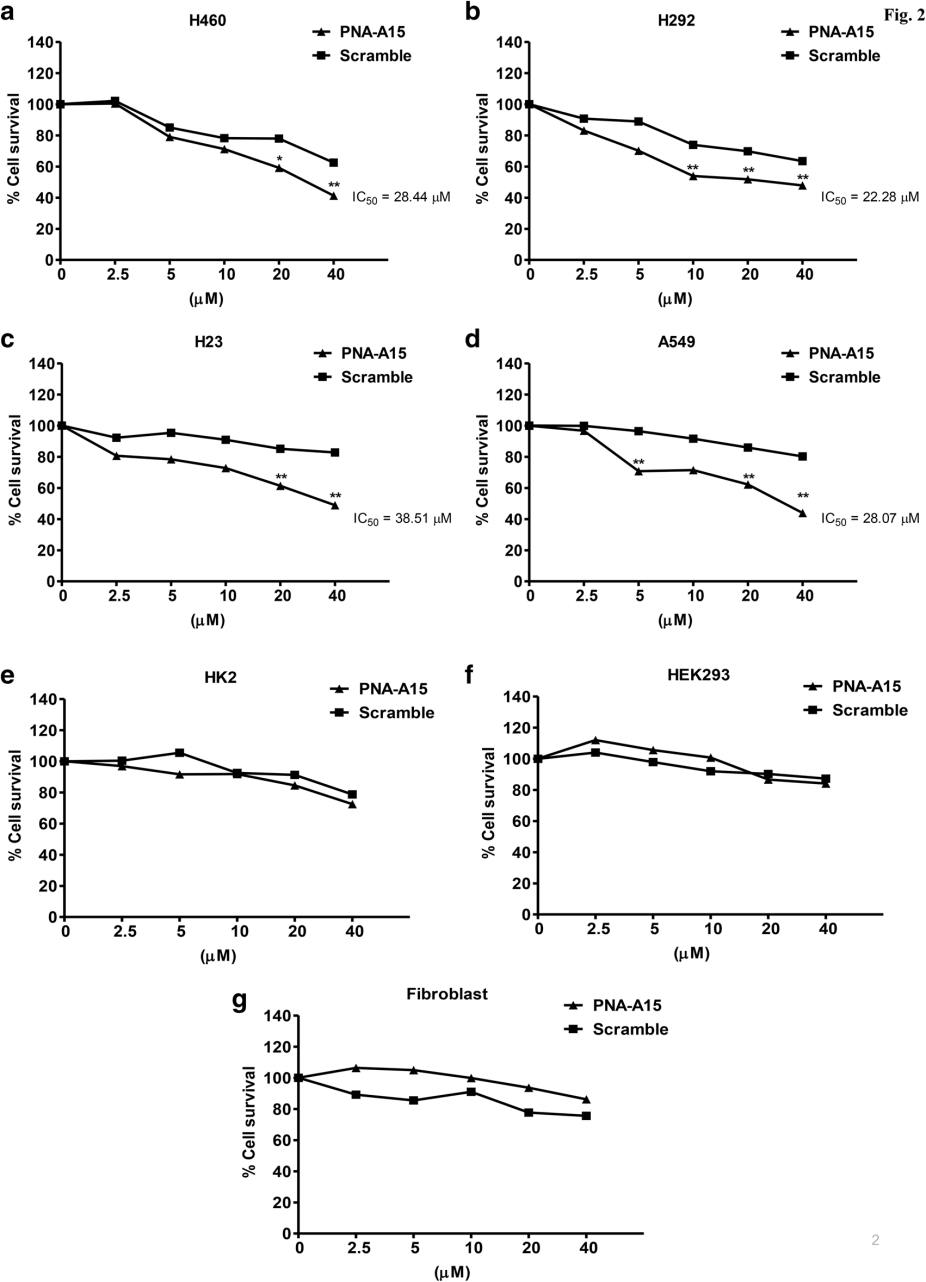
PNA-A15 is not toxic to normal cells but effectively kills lung cancer cells. **a** H460, **b** H292, **c** H23, **d** A549, **e** HK2, **f** HEK293, and **g** PDL fibroblast cells were treated with various concentrations of PNA-A15 (0–40 µM) for 48 h, or scramble, which was used as a control group. Then, cell viability was determined by MTT assay relative to the viability of untreated cells set as 100%. All plots represent the mean ± SD (n = 4). *P < 0.05 vs. untreated cells

Prior to determining the effect of PNA-A15 on CSC properties, the appropriate noncytotoxic concentrations were evaluated. To study the effect of PNA-A15, human NSCLC H460, H292, H23, and A549 cells were treated with various concentrations of compound (0, 2.5, 5, 10, 20, and 40 µM) for 48 h, and then their cell viability was determined by the MTT viability assay. PNA-A15 was found to be nontoxic at concentrations below 5 µM, whereas no cytotoxic effect was found for the scramble control (Fig. 2a–d). Interestingly, PNA-A15 showed no cytotoxic effect on normal cells that contained HK2, HEK293 and PDL fibroblast at all tested concentrations (0–40 µM) (Fig. 2e–g). Thus, nontoxic concentrations of PNA-A15 were further tested for their effects on CSC-like gene expression and phenotypes.

### PNA-A15 altered the transcription of CSC genes containing A-T repeats

**Fig. 3:**
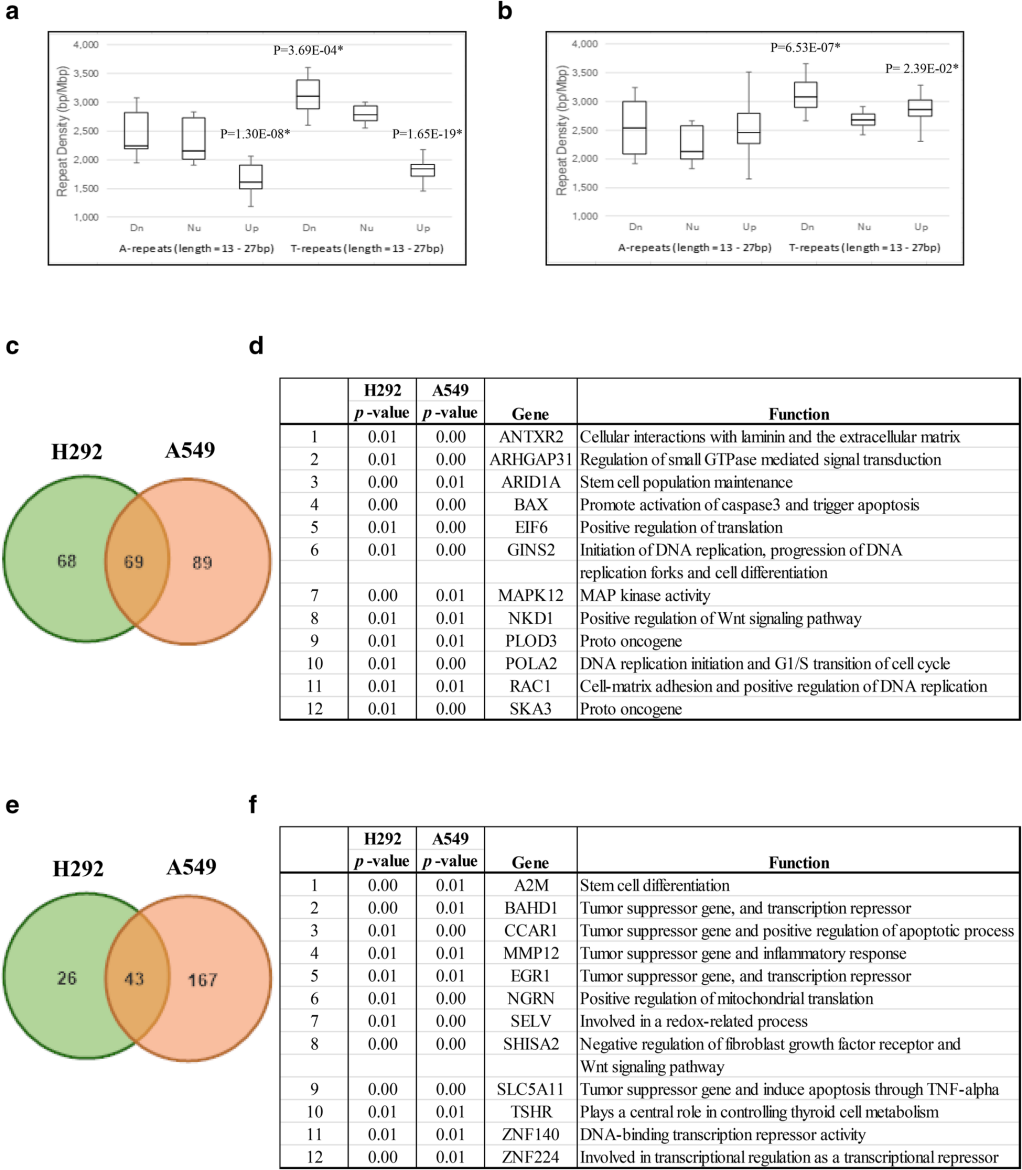
PNA-A15 dysregulated tumour oncogene genes and tumour suppressor genes in lung CSCs. The expression levels of down- and upregulated genes containing A and T repeat sequences of transfected PNA-A15 in spheroid **a** H292 and **b** A549 cancer cell lines were significantly increased and reduced compared with neutral genes. Subjects were divided into experimental and control groups. Dn, Nu, and Up denote downregulated, neutral, and upregulated genes, respectively, in spheroid H292 and A549 cancer cell lines compared with the scramble control in their spheroid cancer cell lines. The repeat density was measured in bp/Mbp at 13–27 bp length. **c** Venn diagram shows overlap between the intersection of downregulated genes in H292 (the left circle) and A549 (the right circle) cell lines. **d** The table of candidate genes determined by biological process from Gene Ontology. The list of genes and their oncogenic function from overlapping between intersecting both cell lines were classified as alphabetical order. **e** Venn diagram shows overlap between the intersection of upregulated genes between H292 (the left circle) and A549 (the right circle) cell lines. **d** The table of candidate genes determined by biological process from Gene Ontology. The list of genes and their tumour-suppressor function from overlapping between intersecting both cell lines were classified as alphabetical order. Student's t-test *P*-values, which are denoted by *P*, indicate that the mean difference between Dn/Up and Nu is statistically significant. All plots show the mean ± SD (n = 3). *P < 0.05 vs. untreated cells

We determined genome-wide gene expression after spheroid cancer cell transfection with PNA-A15. Cells were pretreated with PNA-A15 or scramble PNA oligo for 2 days and then evaluated for spheroid formation in ultralow attachment plates. After that, pretreated cells were seeded at a low density and allowed to form primary spheroids for 7 days. The primary spheroids were then resuspended into single cells, and secondary spheroids were allowed to grow for 14 days. Then, we analysed the expression profiling of A and T repeat genes in the CSC-rich population treated with PNA-A15 compared to their scramble cells. We found that the A repeat densities of upregulated genes were significantly reduced compared with those of neutral genes (*P* values = 1.30E-08), and the T repeat densities of downregulated and upregulated genes were also significantly altered compared with neutral (*P* values = 3.69E-04 and 1.65E-19, respectively) in H292 spheroids. Furthermore, T repeat densities of down- and upregulated genes were also significantly altered compared with neutral genes (*P*-values = 6.53E-07 and 2.39E-02, respectively) in spheroid A549 cells. These results suggest that PNA-A15 significantly alters the expression of genes containing A- and T- repeats in spheroid H292 and A549 cancer cell lines (Fig. 3a, b).

To determine PNA-A15 dysregulated genes in CSCs, we selected candidate genes from overlapping A and T repeat densities of both down- and upregulated genes between the CSC-rich population treated with PNA-A15 and their scramble cells. All genes were classified by a biological process based on Gene Ontology analysis as shown in Fig. 3c and Additional file 1: Table S2. The validation genes were selected based on function and their biological processes. The data represent 69 overlapping genes filtered with a *P *value ≤ 0.01, and we found that PNA-A15 dysregulated the A and T repeat densities of downregulated genes that are related to genes acting as proto-oncogenes and inducing tumorigenesis in both cell lines. PNA-A15 dysregulated the A and T repeat densities of upregulated genes, which act as a tumour suppressor and induce apoptotic processes (Fig. 3e, f and Additional file 1: Table S3).

### PNA-A15 suppresses CSC phenotypes

**Fig. 4:**
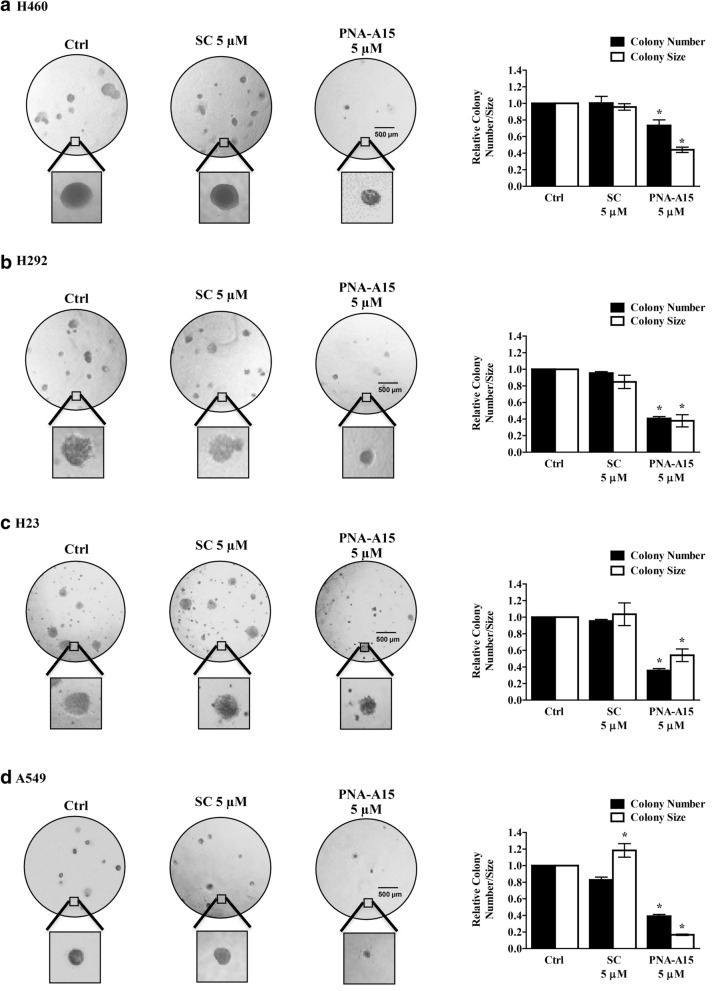
PNA-A15 inhibits the anchorage-independent growth of human lung cancer in H460, H292, H23 and A549 cells. **a** H460, **b** H292, **c** H23, and **d** A549 cells were treated with PNA-A15 or scramble (SC) 5 μM) for 48 h. After treatment, anchorage-independent colony formation was assessed by microscopy (4×) after 14 days, and the relative colony number and size of PNA-A15 or scramble-treated cells were compared to those of untreated controls. All plots present the mean ± SD (n = 3). *P < 0.05 vs. untreated cells

The ability of cancer cells to form 3D spheroids and to grow in anchorage-independent conditions is widely used to assess CSC phenotypes [2, 10]. Therefore, we investigated the role of PNA-A15 in CSC behaviours. Accordingly, the effect of PNA-A15 on the growth and survival of NSCLC-derived cells was evaluated under these conditions. First, H460, H292, H23 and A549 cells were exposed to noncytotoxic concentrations of PNA-A15 for 48 h and subsequently analysed for colony formation in soft agar by recording the relative number and area of colonies after 14 days compared to untreated control or scramble-treated cells (Fig. 4). We found that the PNA-A15-treated cells exhibited a significant decrease in the number and size of colonies compared to the untreated or scramble-treated control cells.

**Fig. 5:**
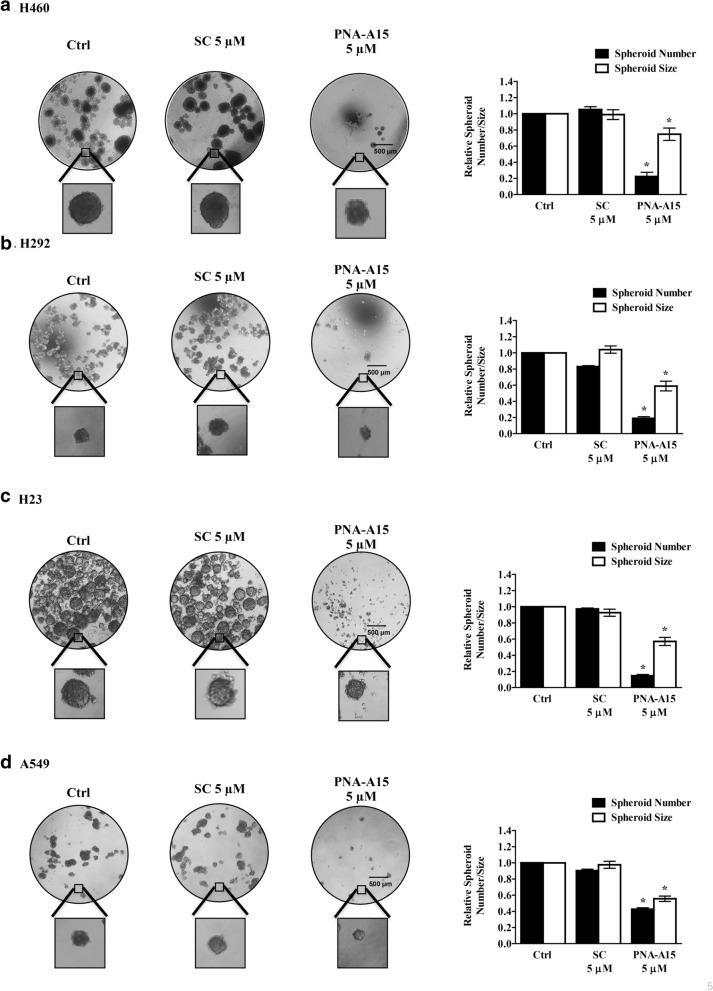
PNA-A15 suppresses CSC-like behaviour of human lung cancer in H460, H292, H23 and A549 cells. **a** H460, **b** H292, **c** H23, and **d** A549 cells were treated with PNA-A15 or scramble (SC) 5 μM) for 48 h. After treatment, cells were grown in a 24-well ultralow attachment plate at a density of 2.5 × 10^3^ cells/well for 7 days to form primary spheroids. These primary spheroids were trypsinized as single cells and replated for 14 days in a 24-well ultralow attachment plate to form secondary spheroids. At day 14, the number and size of the secondary spheroids were determined and imaged using phase-contrast microscopy (Olympus IX51 with DP70). The relative spheroid number and size of PNA-A15- or scramble-treated cells were compared to those of untreated controls. All plots present the mean ± SD (n = 3). *P < 0.05 vs. untreated cells

Then, we evaluated spheroid formation in ultralow attachment plates. We found that treatment of the cells with PNA-A15 significantly inhibited spheroid formation (Fig. 5). These results suggest that PNA-A15 inhibits the growth and survival characteristics of CSCs in NSCLC-derived cells.

**Fig. 6:**
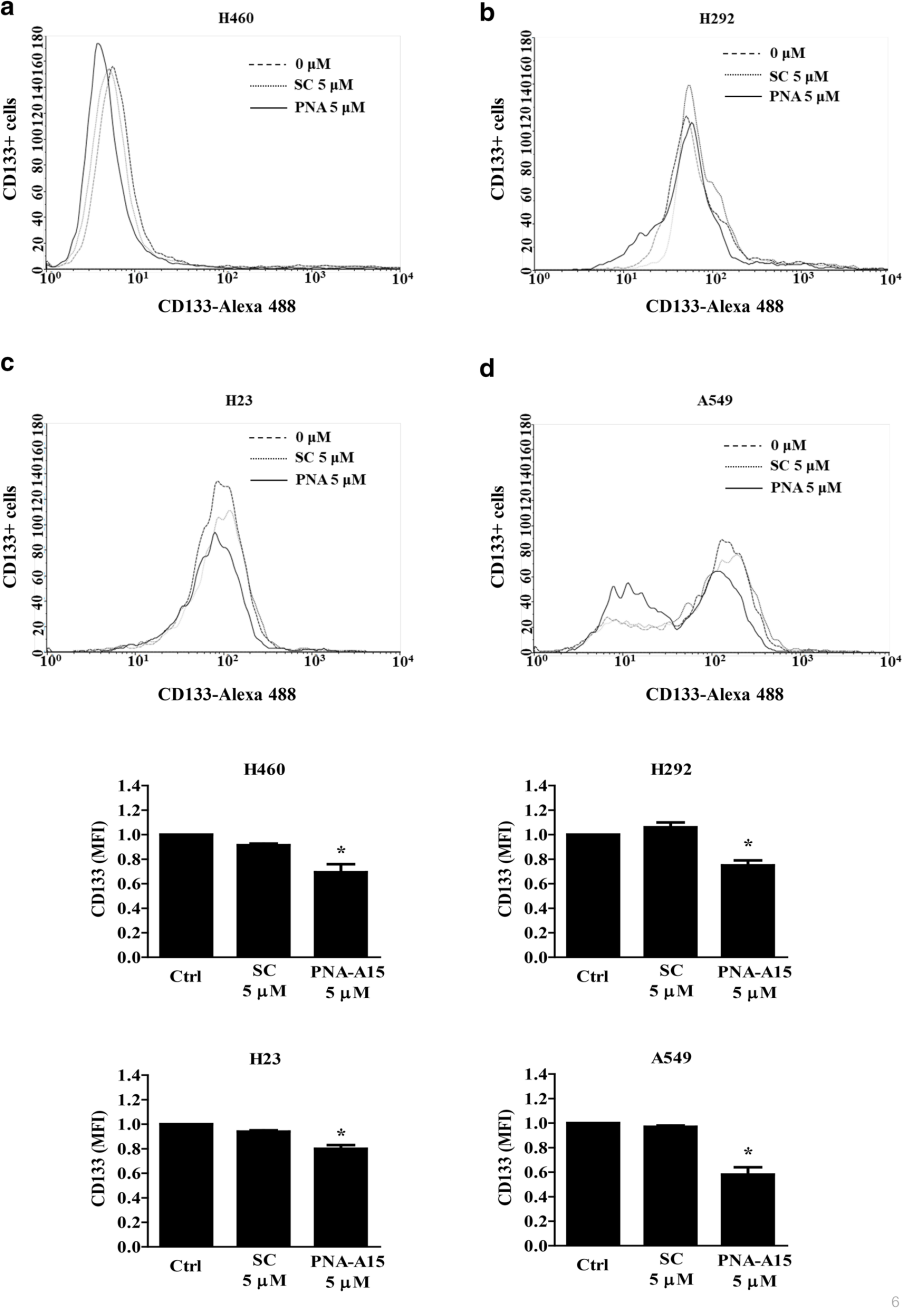
PNA-A15 inhibits the expression of CSC markers. **a** H460, **b** H292, **c** H23, and **d** A549 cells were treated with PNA-A15 or scramble 5 μM for 48 h. CD133 expression levels were assessed by flow cytometry in CSC-enriched cells using anti-CD133 monoclonal antibodies followed by an Alexa Fluor 488-labelled secondary antibody. Data represent the mean ± SD (n = 3). *p < 0.05 versus untreated control

To determine lung CSC markers, immunocytochemistry experiments assessing the expression of CD133 were performed and analysed by flow cytometry. The results showed that PNA-A15 treatment decreased the mean fluorescence intensity of CD133 compared with the untreated or scramble-treated control (Fig. 6).

### PNA-A15 suppresses CSC maintenance in CSC-rich spheroids

**Fig. 7:**
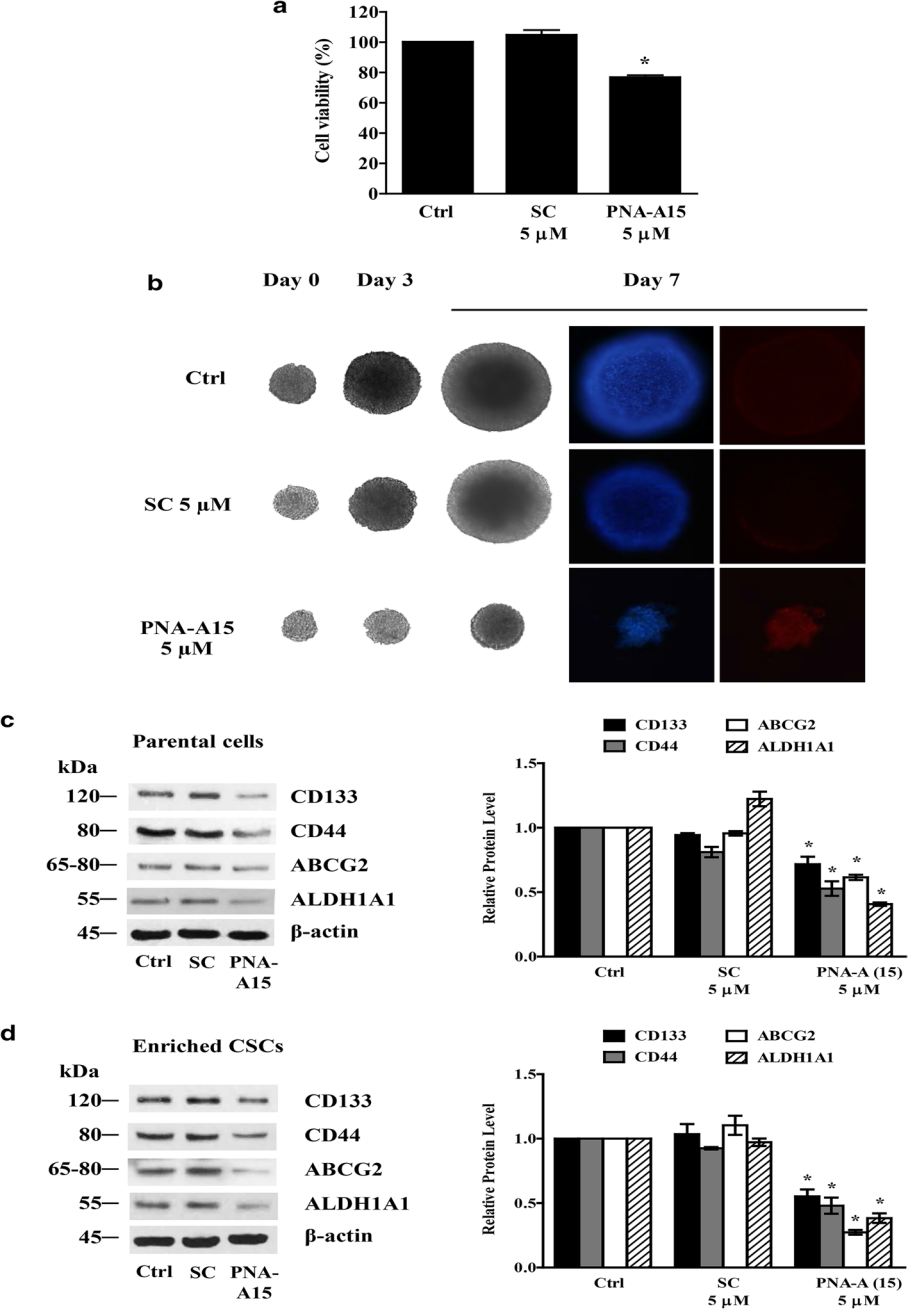
PNA-A15 suppresses the expression of CSC-like phenotypes. **a** Cell viability was assessed using a WST-1 assay, and relative cell viabilities were compared to those of untreated controls. **b** 3D single CSC-enriched populations derived from secondary spheroids of H292 cells were treated with PNA-A15 or scramble 5 μM for 7 days. Spheroid images were captured by microscopy (10 ×) on days 0, 3, and 7 of treatment. Apoptotic and necrotic cell death was assessed by Hoechst 33342/PI costaining. Cells were visualized using fluorescence microscopy (10 ×). **c** Parental H292 cells were treated with PNA-A15 or scramble 5 μM for 48 h, and the expression levels of CD133, CD44, ABCG2 and ALDH1A1 were assessed by Western blotting. **d** Enriched CSC H292 cells were treated with PNA-A15 or scrambled 5 μM for 48 h days, and the expression levels of CD133, CD44, ABCG2 and ALDH1A1 were determined by Western blotting. Blots were reprobed with β-actin to confirm the equal loading of samples. Data represent the mean ± SD (n = 3). *P < 0.05 versus untreated control

We further monitored the effect of PNA-A15 on the maintenance of CSCs in the CSC-rich population. The spheroids were either treated with PNA-A15 or scramble plasmid or untreated for 48 h. Cell viability was then evaluated by WST assay. PNA-A15 significantly decreased the viability of CSCs in H292 cells compared to parental and scrambled cells (Fig. 7a). Every individual spheroid was subsequently treated with PNA-A15 at 5 μM for 0–7 days. We found that PNA-A15 treatment significantly decreased the size of the spheroids and induced apoptosis at days 3 and 7 after treatment as determined by increased chromatin condensation and nuclear fluorescence following Hoechst 33342 and PI staining (Fig. 7b). These results support the above observed inhibitory effect of PNA-A15 on CSCs.

Furthermore, CSC markers, including CD133, CD44, ABCG2 and ALDH1A1, were analysed by Western blot analysis. Figure 7c shows that PNA-A15 caused a significant decrease in CD133, CD44, ABCG2 and ALDH1A1 expression compared with the untreated or scramble-treated control level in parental cells. The results were confirmed in the 3D-CSC-enriched population. The enriched CSC population was treated with PNA-A15 at 5 μM for 48 h, and the expression of CD133, CD44, ABCG2 and ALDH1A1 was determined by Western blot analysis. Figure 7d shows that PNA-A15 treatment reduced CD133, CD44, ABCG2 and ALDH1A1 expression compared with the untreated or scramble-treated control level. These results strongly support the role of PNA-A15 as a multiple gene targeting agent for lung CSCs.

## Discussion

Here, we found that A and T repeats regulate gene expression in CSCs; therefore, PNA-A15 represents a promising targeted therapy agent in lung cancer by downregulating multiple oncogenes and upregulating multiple tumour-suppressor genes. We also revealed that A-T repeat-containing genes that are downregulated in CSCs are involved in CSC morphology, such as BRWD1, FLOT2, SKA3, and TGF-β1. BRWD1 is a transcriptional activator that inhibits cell proliferation by coordinately regulating MYC, and this mechanism plays a role in the self-renewal of CSCs [12]. TGF-β1 is a multifunctional cytokine that plays a critical role in the regulation of CD133 expression. Previous work revealed that TGF-β1 upregulates CD133 expression in hepatocellular carcinoma [13]. FLOT2 is a proto-oncogene that regulates cell migration and invasion in cancer. FLOT2 is involved in cell cycle progression and regulates signalling pathways that are responsible for stemness and induce cadherin binding [14]. FLOT2 is necessary for TGF-β1 and induced epithelial-mesenchymal transition (EMT) in gastric cancer. Suppression of FLOT2 results in decreased cell invasion through repressing TGF-β1-mediated EMT and decreased CSCs [14, 15]. Moreover, SKA3 is a proto-oncogene that induces cell cycle progression. Previous work revealed that SKA3 mRNA expression was elevated and correlated with poor survival outcomes in lung cancer patients [16]. SKA3 acts as an oncogene by directly binding to EGFR and promoting cancer metastasis [16, 17]. Moreover, the SKA3 gene acts as a proto-oncogene and induces tumorigenesis by inhibiting p53 in the apoptotic process. Additionally, p53 regulates the expression of CSC markers. Thus, activating p53 not only increases apoptotic induction in tumour cells but also suppresses CSC self-renewal.

After treatment of PNA-A15 into spheroids of H292 and A549 cells, we found that the expression of many A and T repeat-containing genes changed in both the up- and downregulation directions. CSCs are difficult to therapeutically target because they are inherently resistant to cytotoxic and targeted drugs, and they also evade radiotherapy and immune surveillance [1, 18]. Recent data show that CSC populations underlie significant diversification and plasticity and disguise heterogeneity [18]. Our experiments also demonstrated heterogeneity in the gene expression of CSCs. This heterogeneity prevents the effectiveness of therapy targeting a single target. Therefore, PNA-A15 is a promising targeted therapy in lung cancer by downregulating multiple oncogenes and upregulating multiple tumour suppressor genes. PNA-A15 treatment downregulated A-T repeat-containing genes. Interestingly, many of the genes possess stem cell maintenance and functions, such as the ARID1A, MAPK12, PLOD3, and RAC1 genes [19–22]. Previous studies revealed that ARID1A regulates SOX9 expression, which is a stem cell transcription factor that maintains the self-renewal property of intestinal stem cells [19]. In contrast, the deletion of the ARID1A gene results in the loss of stem cells and increases apoptosis in adult mice [19, 23]. MAPK12 also modulates the stemness of various CSC types by inducing tumorigenesis and cancer aggressiveness [21]. A previous study revealed that MAPK12 significantly increased EMT and promoted CSC regulation in breast cancer [21]. In addition, PNA-A15 interferes with both cancer and CSC phenotypes through the regulation of PLOD3, which is a potent inducer of lung cancer metastasis via the RAS-MAPK pathway in vivo [22]. Moreover, PLOD3 also interacts with STAT3, which is a stem cell transcription factor that maintains the self-renewal property of lung stem cells [22]. PNA-A15 also downregulated the RAC1 gene, which mediated lung tumour growth and increased cell proliferation in vivo. Previous work demonstrated that RAC1 is critically involved in NSCLC migration and lung CSC formation and that RAC1 served as a useful therapeutic target by inhibiting tumour initiation and metastasis of CSCs in lung cancer [20]. Thus, PNA-A15 not only interferes with tumour cells but also suppresses several genes possessing the self-renewal of CSC functions.

PNA-A15 treatment upregulated A-T repeat-containing genes that act as tumour suppressor genes, such as BAHD1, CCAR1, EGR1, and SLC5A11. These genes prevent tumour formation and increase apoptotic processes [24–26]. CCAR1 is a tumour suppressor gene related to p53-induced apoptosis [27]. BAHD1 is a tumour suppressor gene, and this anti-proliferative protein inhibits cell cycle progression from G0/G1 to s phase [24]. EGR1 is a tumour suppressor gene that regulates cell survival by activating the expression of p53 to prevent tumour formation [26]. This role is important because p53 regulates the expression of CSC genes. Thus, activating p53 not only increases apoptotic induction in tumour cells but also suppresses self-renewal of CSCs. CCAR1 mediates the expression of multiple cell cycle regulatory genes and plays a role in cell cycle progression as a transducer of Notch signalling [26]. The Notch signalling pathway is involved in the process of stem cell initiation [28, 29]. In some solid tumours, dysregulation of the Notch signalling pathway is correlated with tumour initiation and increased tumour sphere formation [30]. These findings suggest that PNA-A15 may target the Notch signalling pathway in lung CSCs.

PNA-A15 is not toxic to normal cells but effectively kills cancer cells. Previously, we reported that short A and T repeats (2–9 bp) are more abundant in tissue-specific genes, whereas long A and T repeats (10–30 bp) are more abundant in housekeeping genes [5]. For normal cells, genes controlling differentiation are essential [5, 6]. On the other hand, to maintain the cancer and stem cell phenotypes, cancer cells must prevent full differentiation and use housekeeping genes to play a major role in cell survival. Therefore, long A and T repeats may influence cancer and stem cell survival more than completely differentiated cells.

The stemness of a cancer cell can drive drug resistance through certain mechanisms. For instance, ABCG2, an indicator of cancer stemness, is highly involved in acquiring multidrug resistance to cancer chemotherapeutics such as Cisplatin and Etoposide [31, 32]. In accompany with reduction of CSC, we found that PNA-A15 treatment could reduce ABCG2 expression. Future studies to determine the efficacy of PNA-A15 over drug-resistant phenotype are interesting.

## Conclusions

In conclusion, A-T repeat transcriptional control is essential for CSC biology. Furthermore, interfering with PNA-A15 activity can disrupt CSC growth by dysregulating many genes. This property of PNA-A15 suggests the potential of PNA-A15 in lung CSC treatment to overcome genetic heterogeneity. Moreover, PNA-A15 might be a potential therapeutic drug for many other CSCs in addition to lung cancer.

While PNA-A15 targets CSC, standard chemotherapeutic agents are effective in cancer cells, future study a combinatorial drug between PNA-A15 and standard chemotherapeutic agents may improve treatment efficacy.

## Supplementary Information

**Additional file 1: Table S1.** The lists of down-regulated genes between CSCs and parental cells in H292 and A549 cell lines. The data were filtered with P ≤ 0.05. **Table S2.** The lists of down-regulated genes between PNA-A15 and their scramble in H292 and A549 cell lines. The data were filtered with *P* ≤ 0.05. **Table S3.** The lists of up-regulated genes between PNA-A15 and their scramble in H292 and A549 cell lines. The data were filtered with *P* ≤ 0.05.

## Data Availability

The datasets used and/or analysed during the current study are available from the corresponding author on reasonable request.

## Abbreviations

CSCs: Cancer stem cells
PNA: Peptide nucleic acid
EGFR: Epidermal growth factor receptor
Agos: Agonaute proteins
NSCLC: Non-small cell lung cancer
FBS: Fetal bovine serum
MTT: 3-(4,5-Dimethylthiazol-2-yl)-2,5-diphenyltetrazolium bromide
DMSO: Dimethyl sulfoxide
PI: Propidium iodide
CD133: Cluster of differentiation 133
CD44: Cluster of differentiation 44
ABCG2: ATP-binding cassette super-family G member 2
ALDH1A1: Aldehyde dehydrogenalse 1
HRP: Horseradish peroxidase
SCM: Stem cell media
GEO: Gene Expression Omnibus
EDirect: Entrez Direct
CU-DREAM: Connecting up- and down-regulation expression analysis of microarrays program
IC_50_: The half-maximal inhibition concentration
WST: Water-soluble tetrazolium salt
BRWD1: Bromodomain and WD repeat domain containing 1
FLOT2: Flotillin-2
SKA3: Spindle and kinetochore associated complex subunit 3
TGF-β1: Transforming growth beta 1
EMT: Epithelial-mesenchymal transition
ARID1A: AT-rich interaction domain 1A
MAPK12: Mitogen-activated protein kinase 12
PLOD3: Procollagen-lysine,2-oxoglutarate 5-dioxygenase 3
RAC1: Ras-related C3 botulinum toxin substrate 1
SOX9: SRY-box transcription factor 9
STAT3: Signal transducer and activator of transcription 3
BAHD1: Bromo adjacent homology domain containing 1
CCAR1: Cell division cycle and apoptosis regulator 1
EGR1: Early growth response protein 1
SLC5A11: Solute carrier family 5 member 11
